# Federated discovery and sharing of genomic data using Beacons

**DOI:** 10.1038/s41587-019-0046-x

**Published:** 2019-03-04

**Authors:** Marc Fiume, Miroslav Cupak, Stephen Keenan, Jordi Rambla, Sabela de la Torre, Stephanie O. M. Dyke, Anthony J. Brookes, Knox Carey, David Lloyd, Peter Goodhand, Maximilian Haeussler, Michael Baudis, Heinz Stockinger, Lena Dolman, Ilkka Lappalainen, Juha Törnroos, Mikael Linden, J. Dylan Spalding, Saif Ur-Rehman, Angela Page, Paul Flicek, Stephen Sherry, David Haussler, Susheel Varma, Gary Saunders, Serena Scollen

**Affiliations:** 1 DNAstack, Toronto, Ontario Canada; 2 Global Alliance for Genomics and Health, Toronto, Ontario Canada; 3 European Molecular Biology Laboratory, European Bioinformatics Institute, Wellcome Genome Campus, Hinxton, Cambridge UK; 4 Centre de Regulació Genòmica, Barcelona, Spain; 5 Centre of Genomics and Policy, Department of Human Genetics, McGill University, Montreal, Quebec Canada; 6 Department of Genetics, University of Leicester, Leicester, UK; 7 Genecloud, Sunnyvale, CA USA; 8 ELIXIR Hub, Wellcome Genome Campus, Hinxton, Cambridge UK; 9 Ontario Institute for Cancer Research, Toronto, Ontario Canada; 10 Genomics Institute, University of California at Santa Cruz, Santa Cruz, CA USA; 11 Department of Molecular Life Sciences, University of Zurich, Zurich, Switzerland; 12 SIB Swiss Institute of Bioinformatics, Lausanne, Switzerland; 13 CSC – IT Center for Science Ltd, Espoo, Finland; 14 Broad Institute of MIT and Harvard, Cambridge, MA USA; 15 Wellcome Trust Sanger Institute, Wellcome Genome Campus, Hinxton, Cambridge, UK; 16 National Center for Biotechnology Information, US National Library of Medicine, Bethesda, MD USA

**Keywords:** Genetic testing, Genetics research, Genomics

**To the Editor** — The Beacon Project (https://github.com/ga4gh-beacon/) is a Global Alliance for Genomics & Health (GA4GH)^1^ initiative that enables genomic and clinical data sharing across federated networks. The project is working toward developing regulatory, ethics and security guidance to ensure proportionate safeguards for distribution of data according to the GA4GH-developed “Framework for Responsible Sharing of Genomic and Health-Related Data”^2^. Here we describe the Beacon protocol and how it can be used as a model for the federated discovery and sharing of genomic data.

A Beacon is defined as a web-accessible service that can be queried for information about a specific allele. A user of a Beacon can pose queries of the form “Have you observed this nucleotide (e.g., C) at this genomic location (e.g., position 32,936,732 on chromosome 13)?” to which the Beacon responds with either “yes” or “no.” In this way, a Beacon allows allelic information of interest to be discovered by a remote searcher with no reference to a specific sample or patient, thereby mitigating privacy risks.

In principle, allelic information from any source (or species) can be distributed through a Beacon. For example, a Beacon may serve data from case-level observations, such as genetic variants identified from sequenced samples, or from annotation resources such as variant–disease associations curated from scientific literature. Along with a “yes” response, a Beacon may optionally disclose metadata, including allele frequencies, pathogenicity scores and associated phenotypes, associated with the queried allele. Access to Beacons is securable through institutional systems for authentication and authorization (for example, ELIXIR AAI), allowing hosts to enforce proportionate safeguards for datasets that may be sensitive and consented for use only by trusted individuals and/or for specific purposes.

The Beacon Project is demonstrating the willingness of international organizations to work together to define standards for, and actively engage in, genomic data sharing. Several organizations have ‘lit’ (i.e., implemented) a Beacon, and these have been assembled into a single searchable network. In the years since the project’s inception, over 100 Beacons have been lit by 40 organizations serving over 200 datasets. The datasets served through Beacons are searchable individually or in aggregate—for instance, via the Beacon Network (https://beacon-network.org), a federated search engine across the world’s beacons.

Beacons are a general-purpose protocol for genomics data discovery and have been lit by both large and small organizations, as well as by individuals. This has made available datasets collected from large-scale population sequencing efforts (for example, 1000 Genomes)^3^, clinical diagnostic settings, in silico predictions (for example, PolyPhen-2)^4^, expertly curated or crowd-sourced databases, scientific literature (for example, the Human Genome Mutation Database)^5^ and variant curation efforts (for example, ClinVar)^6^. The International Cancer Genome Consortium^7^ Beacon shares case-level somatic variant observations from over 60 cancer subtypes; the PhenomeCentral^8^ Beacon shares observations from hundreds of clinical cases of undiagnosed and rare genetic diseases; and the BRCA Exchange (https://brcaexchange.org/) Beacon distributes consensus classifications for variants in *BRCA1* and *BRCA2* cataloged by the ENIGMA Consortium^9^, as well as variants collected from other resources as part of the GA4GH BRCA Exchange (https://brcaexchange.org/). The ELIXIR hub (https://elixir-europe.org/) is also integrating Beacon to connect geographically distributed data centers and unify their data access methodologies. This will enable aggregate sharing of allelic observations between sites, a feature that is not yet available through its services. With continued adoption, Beacons will produce a large network of globally searchable genomics datasets that have the potential to unlock new genomics-derived discoveries and applications in medicine.

## Beacon protocol

Many former systems for genomic data sharing have followed a centralized model, wherein data generators deposit information into a single repository, such as the Sequence Read Archive (SRA)^10^. This model requires data generators to transfer whole copies of datasets over the internet, which will become inefficient and expensive as the rate of genomic data acquisition increases. An alternative, federated model for data sharing^1^ requires organizations to host data independently and to interoperate via an agreed-upon technical language. This model removes the inefficiencies of large data transfers and gives host organizations more control over data privacy, security and representation.

For maximal interoperability, a Beacon is designed to be a communication layer that is compatible with any underlying representation of alleles or their annotations. For example, the GA4GH develops a data representation format for genomic variants and annotations, but in practice these data types may be stored in other formats as well (for example, VCF files or relational databases).

Sharing through Beacon is notably different from sharing fully descript data representations for genomic variants (for example, VCF) or annotations (for example, GFF). The Beacon protocol considers levels of data aggregation and obfuscation that can be added onto raw data representations (such as VCF) to convey useful information without explicitly referring to specific samples or individuals.

With these features in mind, the Beacon protocol was designed to be:

- Simple: Beacons can be implemented on top of any underlying variant or variant annotation data store.
- Federated: Beacons can be lit and maintained by individual organizations and assembled into a distributed network.
- General purpose: Beacons can be used to distribute any allelic dataset, including case-level observations or other annotations.
- Aggregative: Beacons provide a boolean answer to whether an allele was observed, possibly aggregated across an entire population, and therefore support deidentification in a way that sharing via VCF files does not.
- Securable: Beacon access can be restricted using institutional security protocols, and authorization schemes can be implemented to respect conditions consented to by patients and/or data owners.

The Beacon API (represented as a RESTful web application) provides a technical specification that a Beacon server must implement. The specification is open-source and available online at https://github.com/ga4gh-beacon/specification.

A Beacon has two available functions: the first lists information about the Beacon, including descriptions of the host organization and specific datasets that it serves; the second queries for the existence of information about specific alleles. Alleles are specified with chromosomal coordinates in addition to reference and alternate bases. Much as in their use in VCF, reference and alternative bases can be used together to specify exact matches for single nucleotide variants (SNVs) and small insertions or deletions. A Beacon responds either “yes” or “no” to signal whether the dataset(s) it serves have information about the queried allele. In the affirmative, a Beacon may optionally disclose metadata describing the observations or annotations associated with the queried allele. An example query and response is shown in Supplementary Fig. 1.

## Reference implementation

To simplify the process of lighting a Beacon, a free, open-source ‘reference implementation’ of the latest specification has been developed.

This implementation can create a public Beacon from a set of VCF files. It may be deployed locally or in a cloud-based environment maintained by a third-party provider (for example, Amazon, Google or Microsoft). Documentation and links to download and run the Beacon reference implementation are available (https://github.com/ga4gh-beacon/). Third-party organizations, such as Cafe Variome, DNAstack and the European Genome-phenome Archive (EGA), also support the ability to light Beacons from genetic variation datasets stored in those systems.

## Beacon security design

In principle, access to Beacons can be secured through any system of authentication or authorization, at the discretion of the host organization. The GA4GH is promoting different levels of data access (open, registered, and controlled) for convenience and for compatibility across its projects. Each so-called ‘access tier’ has distinct visibility and requirements for authorization. For example, ‘open access’ Beacons are accessible to anonymous users of the internet, whereas ‘registered access’ Beacons are accessible to registered users (for example, bona fide researchers and clinicians) who have agreed to a set of conditions of data use^11^.

A Beacon may support one or more access tiers to provide progressive disclosure of increasingly sensitive information (for example, patient phenotypes and clinical information) as users pass through more stringent authentication and authorization checks. For example, tiered access makes it possible for organizations to allow anonymous users to discover the existence of an allelic observation, without the Beacon disclosing more information about it until users identify themselves. The ability for organizations to offer minimal data discovery up front can save substantial time and effort in data access applications when data might not contain relevant data points.

Beacon’s ability to reveal different information at specific access tiers affords genomic data stewards options for distributing allelic information, ranging from fully public to private. Access can be controlled using established authentication and authorization protocols (for example, OpenID Connect and OAuth2.0) to enforce proportionate safeguards for datasets that may be sensitive and/or consented for use only by trusted individuals for specific purposes.

## Attribute disclosure attacks and reidentification

The “yes” response from a Beacon signals the presence of an allele in a dataset comprising possibly many individuals’ genotypes, thereby mitigating risks associated with reidentifying specific individuals. Independent of their technical implementation, Beacon reidentification attempts require prior knowledge of genomic sequence data from the individual (or that of a close relative); they are arguably preceded by more harmful compromises to privacy. However, reidentification can pose additional risks if sensitive attributes about the individual can be inferred from Beacons (for example, HIV status or mental health condition). Such attacks have been characterized as “attribute disclosure attacks using DNA” (ADAD)^12^.

Querying a Beacon for many variants known to exist in a person’s genome could lead to confirmation of that person’s inclusion in a given database, potentially revealing sensitive information about that individual. The ability to reidentify individuals has been examined previously^13^ and recently in the context of Beacons^14^. The power to reidentify an individual whose genotypes are reflected through a Beacon depends on the number of individuals whose data is served, the allele frequency distribution of the pool, the scope of allowed queries (for example, exome versus genome), the type of DNA source (for example, normal tissue versus cancer sample) and the number of times a Beacon is queried. Models for population allele frequencies can be leveraged to reduce the number of queries required in such an attempt, but reidentification is still possible without using allele frequencies if a Beacon can be queried a large number of (for example, 10,000) times.

## Risk mitigation schemes

User agreements, data use policies and technical enforcement of usage quotas can be established to limit the possibility of reidentification and ADAD through Beacons. Organizations are advised to specify terms of use that explicitly prohibit reidentification attempts through the service. When the risk of ADAD is considered too high for data to be distributed publicly, data stewards are encouraged to implement secured access. Compared with public-access tiers, secured-access tiers (either registered or controlled) impose extra social and/or legal disincentives that can help prevent service misuse.

Beacon operators may further specify consent-based data use conditions from a structured set of Consent Codes to impose restrictions indicated by consent of research participants. These Consent Codes, which are general purpose and can be used by genomics data stewards, including Beacon operators, were designed with the purpose of supporting maximum data use and integration while respecting consent permissions^15^. The current set of Consent Codes is provided in Supplementary Table 1.

The ethical, legal and social status of health-related data that are typically considered sensitive in international policy and laws is being examined to provide guidance in aggregating Beacons and in implementing tiered protection of Beacon attributes based on sensitivity^16^. This guidance aims to enable consistent and proportionate provision of data protection for data that are considered more sensitive by individuals and society. Data stewards should consider the sensitivity of attributes used in describing their Beacons, as well as those in the data itself.

Technical provisions can also be used to reduce the statistical power of reidentification attempts. Individual Beacons can be combined to form a single, aggregate Beacon, and direct access to participating Beacons can be blocked. Aggregate beacons contain more data points than any of the individual Beacons while obscuring the origin of the data. As an example, a publicly accessible Beacon named Conglomerate has been lit as an aggregate of multiple independent Beacons.

An information budgeting approach can also be used to thwart reidentification attempts^17^, which rely on accumulating evidence from many queries for alleles carried by a specific individual. The power to reidentify an individual using this technique varies inversely with the frequency of the alleles being queried (i.e., very rare alleles are more revealing than common alleles). By metering the cumulative information disclosure for individuals, Beacons can be configured to restrict access before reidentification is possible within a desired level of statistical confidence.

Beacon is a general-purpose protocol for genomics data discovery, and as such can be used to distribute allelic information from various origins, including sequence observations from patients with known (for example, the International Cancer Genome Consortium)^7^ or unknown (e.g., PhenomeCentral)^8^ diseases, population studies (for example, 1000 Genomes)^3^, in silico predictions (for example, PolyPhen-2)^4^, expertly curated or crowd-sourced databases (for example, BRCA Exchange and ClinVar)^6^, and scientific literature (for example, the Human Genome Mutation Database)^5^. Additional Beacon implementations are ongoing in Europe, mainly through the ELIXIR Beacon project. The deployment of Beacons for select use cases is described below.

## Matchmaking

A major obstacle to discovering the causes of rare diseases is sample size. A single affected family can be enough to identify one or more compelling candidate variants, but pinpointing causal genetic variants frequently requires examining unrelated cases with a variant in the same gene and similar phenotypic presentations. Recently, patient matchmaking has been formalized through efforts such as the Matchmaker Exchange (MME)^18^, in which users who contribute a case to a database within the federated network can find similar cases in other databases within the network.

MME is a secured-access system, requiring that only authorized databases and users can contribute and exchange patient profiles for matching. However, this inherently limits the discoverability of the data, which may dissuade some users having candidate genes or variants they want to match. In addition to implementing the MME API^19^ for patient matchmaking, several organizations within the MME have lit Beacons to serve aggregate views of their clinical datasets more publicly. This allows clinicians with candidate variants to quickly search for existing matches within the MME.

## Sequencing initiatives and archives

Large-scale sequencing initiatives, such as the 100,000 Genomes Project^20^ conducted by Genomics England and the Precision Medicine Initiative^21^, promise to generate vast volumes of genotypic and associated health information. Data from these projects, once shared, help researchers make inferences on the genetic determinants of disease by way of comparative analysis and association studies.

The 1000 Genomes Project^3^, NHLBI Grand Opportunity Exome Sequence Project (https://esp.gs.washington.edu/drupal/), and Exome Aggregation Consortium^22^ are exemplar large-scale initiatives that have shared genotypes from diverse populations through Beacons. As the number and scale of population sequencing efforts expand, a more accurate depiction of global sequence diversity will be available in aggregate through Beacons and the Beacon Network.

In addition, many of the largest genomic archives, such as dbGaP^22^, the European Genome-phenome Archive (https://www.ebi.ac.uk/ega/home) and the European Variation Archive (http://www.ebi.ac.uk/eva), have provided access to variation data through Beacons for some or all of their datasets. These Beacons collectively provide widespread discoverability across a large amount of data. Many of these resources are continually growing with new submissions and thus provide added value for data depositors by simplifying data distribution and unifying their consumption.

## Beacon Network

Beacon represents a simple protocol that, like internet protocols such as HTTP, describes a method for data discovery and exchange between distributed, collaborative systems. Toward developing an ‘internet for genomics’, it is useful to establish a network of protocol adopters and an efficient mechanism for searching across it.

**Fig. 1:**
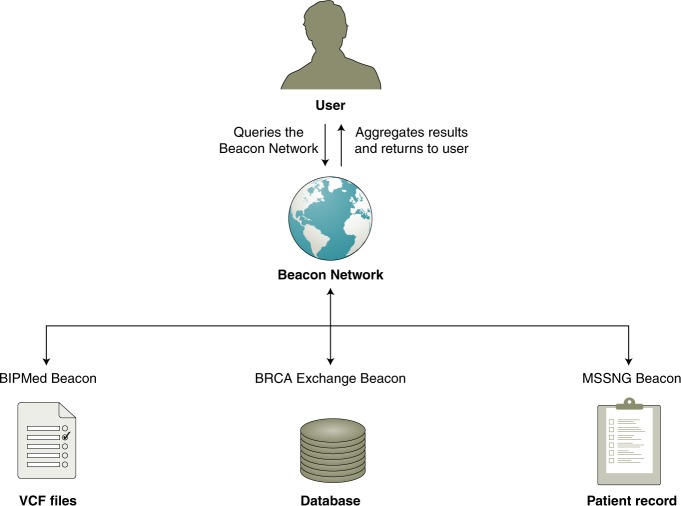
Beacon Network system architecture. The user interacts with the Beacon Network system by asking for information about the existence of a particular genetic mutation. The Beacon Network federates the query across many Beacon instances serving various types of data, such as a variant database, VCF files or patient records. The Beacon Network collects the responses from Beacons and presents aggregated information to the user. BIPMed (http://bipmed.org), the Brazilian Initiative on Precision Medicine, is a population sequencing effort while MSSNG (http://mss.ng) collects sequence information from subjects with autism and their families.

The Beacon Network is a directory and search engine for Beacons. Although individual Beacons answer the question “Have you observed this allele?”, the Beacon Network answers the question “Who has observed this allele?”. The Beacon Network serves as a powerful, convenient and real-time genomic data distribution channel through which users can discover the existence of alleles of interest and be directed to host organizations who have observed them. A schematic of the Beacon Network as a global federated network for genomic information discovery is shown in Fig. 1.

The Beacon Network is accessible either through its website or programmatically through an API, and enables fast, simultaneous search of hundreds of datasets from hundreds of thousands of individuals already served through Beacons worldwide.

Beacons can be freely registered to the Beacon Network and can be searched independently or in aggregate with other connected Beacons. The Beacon Network has received over 1.5 million queries in the three years since its launch. The value of datasets connected to the Beacon Network increases as more Beacons join, particularly for comparative applications like rare disease and donor matching.

## Conclusions and perspectives

The first version of the Beacon Project has validated the feasibility of a globally federated system for genomic data sharing. The conceptual and technical simplicity of the discovery question, “Have you observed this allele?”, enabled rapid and widespread adoption, and this has served to provide practical feedback for the GA4GH to continue to advance its best practices by holistically addressing regulatory, security and technical aspects of global genomics data sharing. However, the narrow focus of the initial Beacon question limits its utility to support other closely related use cases, and successive iterations of the protocol are planned to enable coverage of these.

Future extensions to the Beacon protocol may include the following:

- Support for discovering complex genomic alterations, including copy number variations (CNVs) and somatic copy number alterations (CNAs), which are major contributors to both inter-individual variation and disease susceptibility and prominent features of the oncogenomic mutation landscape;
- Integration of non-genomics data in queries, including the ability to discover similar cases on the basis of associated metadata;
- Support for quantitative attributes in responses (for example, allele frequencies) to facilitate statistical analyses that combine information disclosed through multiple Beacons;
- Handoff to services by which users may access additional information about a queried variant.

The development of data-rich extensions to the Beacon protocol will leverage the expertise of GA4GH members and stakeholders to iteratively design and evaluate the technical, privacy and security considerations in evolving Beacons to enable unprecedented access to genomics and clinical datasets through a global, federated ecosystem.

## Supplementary information

Supplementary Text and Figures Supplementary Figure 1 and Supplementary Table 1
