# Publisher Correction: Federated discovery and sharing of genomic data using Beacons

**DOI:** 10.1038/s41587-019-0094-2

**Published:** 2019-03-20

**Authors:** Marc Fiume, Miroslav Cupak, Stephen Keenan, Jordi Rambla, Sabela de la Torre, Stephanie O. M. Dyke, Anthony J. Brookes, Knox Carey, David Lloyd, Peter Goodhand, Maximilian Haeussler, Michael Baudis, Heinz Stockinger, Lena Dolman, Ilkka Lappalainen, Juha Törnroos, Mikael Linden, J. Dylan Spalding, Saif Ur-Rehman, Angela Page, Paul Flicek, Stephen Sherry, David Haussler, Susheel Varma, Gary Saunders, Serena Scollen

**Affiliations:** 1 DNAstack, Toronto, Ontario Canada; 2 Global Alliance for Genomics and Health, Toronto, Ontario Canada; 3 European Molecular Biology Laboratory, European Bioinformatics Institute, Wellcome Genome Campus, Hinxton, Cambridge UK; 4 Centre de Regulació Genòmica, Barcelona, Spain; 5 Centre of Genomics and Policy, Department of Human Genetics, McGill University, Montreal, Quebec Canada; 6 Department of Genetics, University of Leicester, Leicester, UK; 7 Genecloud, Sunnyvale, CA USA; 8 ELIXIR Hub, Wellcome Genome Campus, Hinxton, Cambridge UK; 9 Ontario Institute for Cancer Research, Toronto, Ontario Canada; 10 Genomics Institute, University of California at Santa Cruz, Santa Cruz, CA USA; 11 Department of Molecular Life Sciences, University of Zurich, Zurich, Switzerland; 12 SIB Swiss Institute of Bioinformatics, Lausanne, Switzerland; 13 CSC – IT Center for Science Ltd, Espoo, Finland; 14 Broad Institute of MIT and Harvard, Cambridge, MA USA; 15 Wellcome Trust Sanger Institute, Wellcome Genome Campus, Hinxton, Cambridge, UK; 16 National Center for Biotechnology Information, US National Library of Medicine, Bethesda, MD USA

**Keywords:** Genetic testing, Genetics research, Genomics

Correction to: *Nature Biotechnology* 10.1038/s41587-019-0046-x, published online 4 March 2019.

In the version of this article initially published, Lena Dolman’s second affiliation was given as Wellcome Trust Sanger Institute, Wellcome Genome Campus, Hinxton, Cambridge, UK. The correct second affiliation is Ontario Institute for Cancer Research, Toronto, Ontario, Canada. The error has been corrected in the HTML and PDF versions of the article.

