# Supplementary material for: Federated discovery and sharing of genomic data using Beacons
Source: Nat Biotechnol. 2019 Mar 4;37(3):220–4. doi: 10.1038/s41587-019-0046-x (PMC6728157; doi:10.1038/s41587-019-0046-x)
Supplement: Supplementary file 1 — Supplementary Figure 1 and Supplementary Table 1 [file 41587_2019_46_MOESM1_ESM.pdf]

nature biotechnology

SUPPLEMENTARY INFORMATION

https://doi.org/10.1038/s41587-019-0046-x

In the format provided by the authors and unedited.

Federated discovery and sharing of genomic data using Beacons

Marc Fiume[1]*, Miroslav Cupak[1], Stephen Keenan[2,3], Jordi Rambla[4], Sabela de la Torre[4], Stephanie O. M. Dyke[5], Anthony J. Brookes[6], Knox Carey[7], David Lloyd[8], Peter Goodhand[2,9], Maximilian Haeussler[10], Michael Baudis[11,12], Heinz Stockinger[12], Lena Dolman[2,9], Ilkka Lappalainen[3,13], Juha Törnroos[13], Mikael Linden[13], J. Dylan Spalding[3], Saif Ur-Rehman[3], Angela Page[2,14], Paul Flicek[3,15], Stephen Sherry[16], David Haussler[10], Susheel Varma[8], Gary Saunders[8] and Serena Scollen[8]

[1]DNAstack, Toronto, Ontario, Canada. [2]Global Alliance for Genomics and Health, Toronto, Ontario, Canada. [3]European Molecular Biology Laboratory, European Bioinformatics Institute, Wellcome Genome Campus, Hinxton, Cambridge, UK. [4]Centre de Regulació Genòmica, Barcelona, Spain. [5]Centre of Genomics and Policy, Department of Human Genetics, McGill University, Montreal, Quebec, Canada. [6]Department of Genetics, University of Leicester, Leicester, UK. [7]Genecloud, Sunnyvale, CA, USA. [8]ELIXIR Hub, Wellcome Genome Campus, Hinxton, Cambridge, UK. [9]Ontario Institute for Cancer Research, Toronto, Ontario, Canada. [10]Genomics Institute, University of California at Santa Cruz, Santa Cruz, CA, USA. [11]Department of Molecular Life Sciences, University of Zurich, Zurich, Switzerland. [12]SIB Swiss Institute of Bioinformatics, Lausanne, Switzerland. [13]CSC – IT Center for Science Ltd, Espoo, Finland. [14]Broad Institute of MIT and Harvard, Cambridge, MA, USA. [15]Wellcome Trust Sanger Institute, Wellcome Genome Campus, Hinxton, Cambridge, UK. [16]National Center for Biotechnology Information, US National Library of Medicine, Bethesda, MD, USA. *e-mail: marc@dnastack.com

Supplementary Figure 1: Beacon query and response for BRCA Exchange Beacon.

Query


```
{
 "referenceName":"13",
 "start":32936731,
 "referenceBases":"G",
 "alternateBases":"C",
 "assemblyId":"GRCh37",
 "datasetIds": [
   “brca”
 ],
 "includeDatasetResponses": true
}
```


Response


```
{
 "beaconId":"brca-exchange",
 "exists": true,
 "alleleRequest": {
   "referenceName":"13",
   "start":32936731,
   "referenceBases":"G",
   "alternateBases":"C",
   "assemblyId":"GRCh37",
   "datasetIds": [
     “brca”
   ],
   "includeDatasetResponses": true
 }
 "datasetAlleleResponses": [
   {
     "datasetId":"brca",
     "exists":true,
     "info":{
       "Clinical_significance_citations":"PMID:23108138",
       "HGVS_cDNA":"c.7878G>C",
       "Date_last_evaluated":"8/10/15",
       "HGVS_protein":"p.(Trp2626Cys)",
       ...
       "Condition_category":"Disease",
       "Comment_on_clinical_significance":"IARC class based on posterior probability from multifactorial
likelihood analysis, thresholds for class as per Plon et al. 2008 (PMID: 18951446). Class 5 based on
posterior probability = 1",
       "Gene_symbol":"BRCA2",
       "Clinical_significance":"Pathogenic",
       "Allele_origin":"Germline",
       "Collection_method":"Curation",
       "Condition_ID_value":"BREAST-OVARIAN CANCER, FAMILIAL, SUSCEPTIBILITY TO, 2; BROVCA2 (612555)"
     }
   }
 ]
}
```

Supplementary Table 1: Consent Codes

Name	Abbreviation	Description
**Primary Categories**		
no restrictions	NRES	No restrictions on data use.
general research use and clinical care	GRU(CC)	For health/medical/biomedical purposes and other biological research, including the study of population origins or ancestry.
health/medical/biomedical research and clinical care	HMB(CC)	Use of the data is limited to health/medical/biomedical purposes, does not include the study of population origins or ancestry.
disease-specific research and clinical care	DS-[XX](CC)	Use of the data must be related to [disease].
population origins/ancestry research	POA	Use of the data is limited to the study of population origins or ancestry.
**Secondary Categories**		
other research-specific restrictions	RS-[XX]	Use of the data is limited to studies of [research type] (e.g., pediatric research).
research use only	RUO	Use of data is limited to research purposes (e.g., does not include its use in clinical care).
no “general methods” research	NMDS	Use of the data includes methods development research (e.g., development of software or algorithms) ONLY within the bounds of other data use limitations.
genetic studies only	GSO	Use of the data is limited to genetic studies only (i.e., no research using only the phenotype data).
**Requirements**		

not-for-profit use only	NPU	Use of the data is limited to not-for-profit organizations.
publication required	PUB	Requestor agrees to make results of studies using the data available to the larger scientific community.
collaboration required	COL-[XX]	Requestor must agree to collaboration with the primary study investigator(s).
return data to database/resource	RTN	Requestor must return derived/enriched data to the database/resource.
ethics approval required	IRB	Requestor must provide documentation of local IRB/REC approval.
geographical restrictions	GS-[XX]	Use of the data is limited to within [geographic region].
publication moratorium/embargo	MOR-[XX]	Requestor agrees not to publish results of studies until [date].
time limits on use	TS-[XX]	Use of data is approved for [x months].
user-specific restrictions	US	Use of data is limited to use by approved users.
project-specific restrictions	PS	Use of data is limited to use within an approved project.
institution-specific restrictions	IS	Use of data is limited to use within an approved institution.
